# Reducing the impact of insulin sensitivity variability on glycaemic outcomes using separate stochastic models within the STAR glycaemic protocol

**DOI:** 10.1186/1475-925X-13-43

**Published:** 2014-04-16

**Authors:** Felicity Thomas, Christopher G Pretty, Liam Fisk, Geoffrey M Shaw, J Geoffrey Chase, Thomas Desaive

**Affiliations:** 1 Department of Mechanical Engineering, University of Canterbury, Private Bag 8140, Christchurch, New Zealand; 2 Department of Intensive Care, Christchurch Hospital, Private Bag 4710, Christchurch, New Zealand; 3 Thermodynamics of Irreversible Processes, Institute of Physics, Allée du 6 Août, 17 (Bât B5), B4000 Liège, Belgium

**Keywords:** Insulin sensitivity, Intensive care, Glycaemia, Model-based control

## Abstract

**Background:**

The metabolism of critically ill patients evolves dynamically over time. Post critical insult, levels of counter-regulatory hormones are significantly elevated, but decrease rapidly over the first 12–48 hours in the intensive care unit (ICU). These hormones have a direct physiological impact on insulin sensitivity (SI). Understanding the variability of SI is important for safely managing glycaemic levels and understanding the evolution of patient condition. The objective of this study is to assess the evolution of SI over the first two days of ICU stay, and using this data, propose a separate stochastic model to reduce the impact of SI variability during glycaemic control using the STAR glycaemic control protocol.

**Methods:**

The value of SI was identified hourly for each patient using a validated physiological model. Variability of SI was then calculated as the hour-to-hour percentage change in SI. SI was examined using 6 hour blocks of SI to display trends while mitigating the effects of noise. To reduce the impact of SI variability on achieving glycaemic control a new stochastic model for the most variable period, 0–18 hours, was generated. Virtual simulations were conducted using an existing glycaemic control protocol (STAR) to investigate the clinical impact of using this separate stochastic model during this period of increased metabolic variability.

**Results:**

For the first 18 hours, over 80% of all SI values were less than 0.5 × 10^-3^ L/mU.min, compared to 65% for >18 hours. Using the new stochastic model for the first 18 hours of ICU stay reduced the number of hypoglycaemic measurements during virtual trials. For time spent below 4.4, 4.0, and 3.0 mmol/L absolute reductions of 1.1%, 0.8% and 0.1% were achieved, respectively. No severe hypoglycaemic events (BG < 2.2 mmol/L) occurred for either case.

**Conclusions:**

SI levels increase significantly, while variability decreases during the first 18 hours of a patients stay in ICU. Virtual trials, using a separate stochastic model for this period, demonstrated a reduction in variability and hypoglycaemia during the first 18 hours without adversely affecting the overall level of control. Thus, use of multiple models can reduce the impact of SI variability during model-based glycaemic control.

## Introduction

The metabolism of critically ill patients evolves dynamically over time [1-3]. Post critical insult, plasma concentrations of counter-regulatory hormones such as cortisol, glucagon, growth hormone, and the catecholamines are significantly elevated, but decrease rapidly over the first 12–48 hours in the intensive care unit (ICU) [2-5]. These hormones have a direct physiological impact on insulin sensitivity (SI) [3]. Therefore, SI is likely to be lowest during the first 12–48 hours in the ICU and increase over time [6-8]. Understanding the variability of SI, over hours and days, is important for safely and effectively managing glycaemic levels, as well as for understanding the evolution of patient condition.

Bagshaw et al. [9] reported associations between mortality, hypoglycaemia, and variability during the first 24 hours of ICU stay. Several other studies [10-13] have also shown that glycaemic variability is independently associated with mortality in critically ill patients. Accurate predictions of SI and its variability would thus enable safer and more effective glycaemic control.

The STAR (Stochastic TARgeted) glycaemic control protocol, has been used in Christchurch Hospital ICU since 2012 [14]. This protocol uses a physiological glucose-insulin system model coupled with stochastic models of SI variability [15,16] to determine the most appropriate insulin and nutrition treatment combinations. SI is an important consideration in clinical blood glucose (BG) control as it captures the overall glycaemic response of a body to exogenous insulin and nutrition inputs. This measure of glycaemic response to exogenous inputs is particularly important for STAR which aims to minimise the risk of hypoglycaemia by directly accounting for likely variability of SI.

We propose that by using multiple stochastic models, specific to patient condition, performance of the STAR protocol could be enhanced by directly accounting for periods of increased SI variability. With only a small alteration to the controller, use of different stochastic models based on time or diagnosis could allow the existing STAR protocol to achieve more accurate control and/or reduce hypoglycaemia. The objective of this study is to assess the evolution of SI in both magnitude and variability over the first few days of ICU stay, and thus propose and test appropriate additional stochastic models to reduce the impact of SI variability on achieving glycaemic control within the STAR protocol.

## Subjects and methods

### Patients

This study used data from 371 patients admitted to the Christchurch Hospital ICU between 2005 and 2007 and treated with the SPRINT (Specialised Relative Insulin Nutrition Tables) glycaemic control protocol [17]. The variability study and the virtual trials were conducted on two different sub sets of patients from this cohort. Table 1 presents a summary of the cohort demographics. The Upper South Regional Ethics Committee, New Zealand granted approval for the audit, analysis and publication of this data.

**Table 1:** Cohort and Sub-cohort summary statistics

	**All patients**	**Patients that started SPRINT within 18 hrs of ICU admission (used to generate Stochastic Model)**	**Patients that started SPRINT with in 12 hrs of ICU admission and continued for at least 24 hrs (used to test separate stochastic model)**
**Number**	371	287	164
**Age (years)**	65 [49–74]	65 [55–74]	65 [56–74]
**Gender (M/F)**	236/135	181/106	102/62
**APACHE II score**	18 [15–24]	18 [14–24]	19 [16–25]
**APACHE II ROD (%)**	26 [13–49]	26 [13–44]	32 [17–52]
**Operative/Non- operative**	170/201	143/144	66/98
**Diabetic status (T1DM/T2DM)**	14/49	13/44	10/22
**Hospital Mortality**	16%	22%	25%
**ICU length of stay (hrs)**	98 [41–251]	72 [26–184]	142 [70–308]

### Analysis of SI variability

#### *Patients*

The analysis of SI variability was performed on a sub-cohort of 164 patients from the SPRINT study [17]. These particular patients were included because SPRINT was commenced within 12 hours of ICU admission and continued for at least 24 hours, ensuring that enough data was available to accurately assess the change in SI during the first 24 hours of glycaemic control.

#### *Methods*

SI was identified hourly for each patient using the validated Intensive Control Insulin-Nutrition-Glucose (ICING) model [18]. This is the same model used in the model-based STAR protocol [14]. Variability of SI was calculated as the hour-to-hour percentage change in SI (Δ%SI):

(1) $$\Delta \%\mathrm{SI}=100\frac{\left(SI_{k+1}-SI_{k}\right)}{SI_{k}}$$

The use of percentage change, rather than absolute change, normalises the metric so patients with differing SI levels can be compared fairly.

Bagshaw et al. [9] reported an association between both hypoglycaemia and variability with mortality during the first 24 hours of ICU stay. Thus, the acute evolution of SI over the first day using 6-hour blocks was analysed. For the cohort analysis, SI and Δ%SI data from all patients was grouped into each appropriate time-block. Median values for each time-block were calculated for comparison to the previous block, thus capturing overall cohort changes over time in level and hour-to-hour variability.

For the per-patient analysis, the median value of SI and the interquartile range (IQR) of Δ%SI were calculated for each patient, for each time-block. The IQR captures the width of degree of variability of a given patient within each 6-hour block. Thus, a reduction in the IQR of Δ%SI over time would indicate a reduction in hour-to-hour variability for a given patient.

Analyses were based on the time spent on the SPRINT protocol, rather than time spent in the ICU, to ensure sufficient insulin and nutrition data to accurately identify an hourly value of SI [19]. Therefore, day 1 comprises the first 24 hours of SPRINT. However, patients were only included in this sub-cohort if they commenced SPRINT within 12 hours of ICU admission. Thus, this data is representative of the first day of ICU stay. The median delay between admission and commencement of SPRINT for this cohort was 1.9 hours and 81% of the cohort (N = 134) had commenced SPRINT within 6 hours of admission, which is well within the reported 24 hour increased variability window [9].

SI levels and variability were compared using cumulative distribution functions (CDFs) and non-parametric statistics. All distributed data was compared using the Wilcoxon rank-sum test (Mann–Whitney U-test), except for SI variability results. SI variability was compared using the Kolmogorov-Smirnov test as it has greater power to detect differences in the shape of distributions when median values are similar. P-values < 0.05 were considered statistically significant.

### Virtual trial simulation and star protocol

#### *Patients*

For this virtual trial, and based on the results of the variability analysis, a new stochastic model based only on data collected during the first 18 hours of patient stay in the ICU was used. Thus, data from 287 patients who commenced SPRINT within 18 hour of ICU admission was used. Specifically, if a patient commenced SPRINT 13 hours after ICU admission, only data from the first 5 hours of glycaemic control would contribute to the *0*–*18 hours stochastic model*, and the remainder to the *18+ hours model*. These 287 patients where then used in simulation to investigate the potential benefits of using two stochastic models.

#### *Methods*

The virtual trial simulation method used in this study is described in detail by Chase et al. [20]. This method involves using the SI profile of patients, identified from actual clinical data, as the underlying basis for virtual patients. During a virtual trial, a control algorithm is used to select an insulin and nutrition intervention, and the known SI profile is used to simulate the resulting BG profile and take a virtual BG measurement at the next intervention time. Prior work has validated this methodology [20], and this information can be used with the STAR controller to assess the benefits of using separate stochastic models. Cohort statistics such as percentage time in a target band and BG distributions achieved highlight the potential effects of changes to the control algorithm.

**Figure 1:**
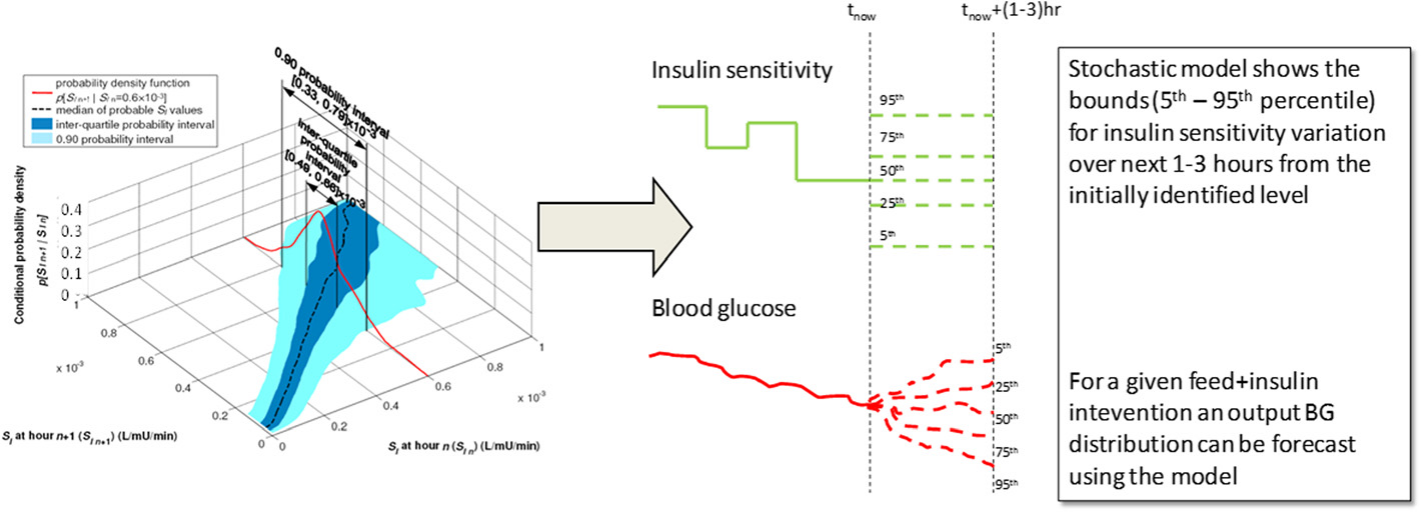
**Schematic of the stochastic model showing the percentiles (5th - 95th) for insulin sensitivity variation for the forthcoming 1–3 hours (n + 1).** For a feed and insulin intervention an output BG distribution can be forecast using the physiological glucose – insulin model.

The STAR protocol recommends insulin and nutrition interventions based on the predicted BG response over 1–3 hour intervals using a forecasted SI from the stochastic model, as shown in Figure 1 [14]. The STAR protocol targets a BG range by maximising the likelihood of achieving that range, given constraints such as acceptable risk of hypoglycaemia and limitations on insulin and nutrition delivery. If BG is stable, the STAR protocol allows 2 and 3-hour BG measurement options. The STAR protocol is described in detail by Fisk et al. [14,21]. For this analysis, the *in-silico* STAR controller always selects the longest available measurement interval, to obtain the best balance between the level of control and expected nurse workload.

Currently, the STAR protocol uses a single stochastic model for all patients, for the entire duration of control (the *general model*) [21]. Hence, this method has no temporal variability. Based on the results of the variability analysis presented in this study, a specific model was generated from data collected during the first 18 hours of patient stay in the ICU, and used only during this period. An *18+ hour model* covers all subsequent time. During virtual trial simulation, if a virtual-patient commences control 13 hours after admission, only the first 5 hours would be controlled using the *0*–*18 hour stochastic model*.

This study compares outcome glycaemia from virtual trials during the first 18 hours of ICU stay. The *18+ hour stochastic model* is sufficiently similar to the *general model* that comparing results from virtual trials using these two models would show no appreciable difference. Further, by analysing and presenting only the data from the first 18 hours of patient stay (4398 hours for the cohort), changes to the outcome glycaemia are clear, as they are not overwhelmed by the much larger quantity of data post-18 hours (27792 hours for the cohort).

## Results

### Insulin sensitivity level and variability

In addition to the actual insulin sensitivity, the SI parameter also captures any sensor noise and un- or under-modelled processes, such as variability in hepatic glucose output. Therefore, Insulin Sensitivity was examined using 6-hour blocks of SI to display trends while reducing any noise effects through averaging. SI increased over each 6-hour block during the first 24 hours of data, while SI variability decreased over this period. The top half of Table 2 displays the percentage increase in median SI for both the overall cohort and per-patient analyses. It is clear from this table that the first 18 hours result in the largest increase in SI level.

**Table 2:** The top half of the table displays the increasing cohort and per patient median insulin sensitivity over 6hr blocks and the second half of the table shows the reductions in the IQR and median per-patient hour to hour percentage insulin sensitivity over time

**SI level analysis**	**Cohort analysis**		**Per-patient analysis**	
	**% increase at median**	**p-value**	**% increase at median**	**p-value**
**0-6 vs 6–12 hrs**	42	< 0.0001	40	0.0007
**6-12 vs 12–18 hrs**	28	<0.0001	26	0.0123
**12-18 vs 18–24 hrs**	1	0.0335	3	0.4822
**18-24 vs 24–48 hrs**	9	0.0428	7	0.2873
**Variability analysis**	**% Change of IQR**	**p-value**	**% Change at median**	**p-value**
**0-6 vs 6–12 hrs**	-36	0.0092	-39	<0.0001
**6-12 vs 12–18 hrs**	-24	0.0806	-29	0.0794
**12-18 vs 18–24 hrs**	1	0.0806	-9	0.1029
**18-24 vs 24–48 hrs**	-19	0.0998	-18	0.0467

P-values calculated using Kolmogorov-Smirnov test for cohort comparisons and Wilcoxon rank-sum for per-patient comparisons.

**Figure 2:**
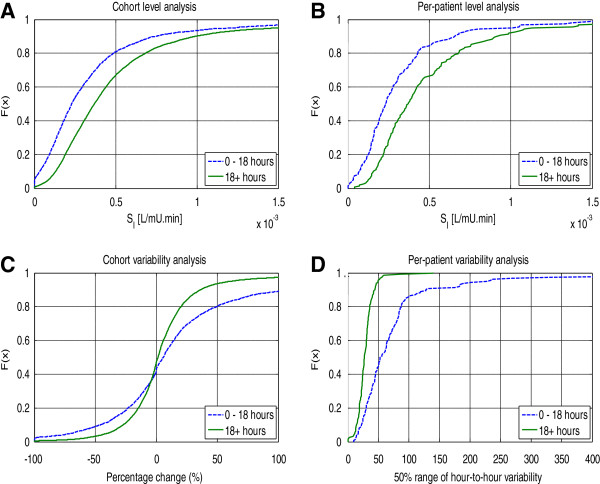
**Cumulative distributions for Insulin sensitivity level and variability by cohort and per patient for the first 0 -18hours compared to the rest of a patients stay. (A)** insulin sensitivity level by cohort **(B)** insulin sensitivity level per patient **(C)** hour-to-hour variability of insulin sensitivity by cohort **(D)** hour-to-hour variability of insulin sensitivity per patient.

The bottom half of Table 2 shows the reduction in SI variability over time. These values represent the percentage change to the IQR-width of Δ%SI and the shift at the median of per-patient IQR-widths (see Figure 2), for the cohort and per-patient analyses, respectively. Again, the largest change is seen over the first 18 hours. Past 18 hours, both variability and magnitude of SI are similar to that seen on days 2, 3 and 4 (results not presented here) [8].

Figure 2 summarises the difference in SI and variability observed during the first 18 hours of patient data compared to the rest of their stay. For data collected during the first 18 hours of stay, more than 80% of all SI values were less than 0.5 × 10^-3^ L/mU.min, compared to 65% for >18 hours. Both per patient panels (B and D) show the same trends as the whole-cohort results, with the *0*–*18 hour model* exhibiting a higher SI level (80% < 0.4 × 10^-3^ L/mU.min) and greater variability. As the first 18 hours produced the greatest increase in SI level and reduction in variability, this period was selected as the most likely to see benefit from using a separate stochastic model.

### Virtual trials

**Figure 3:**
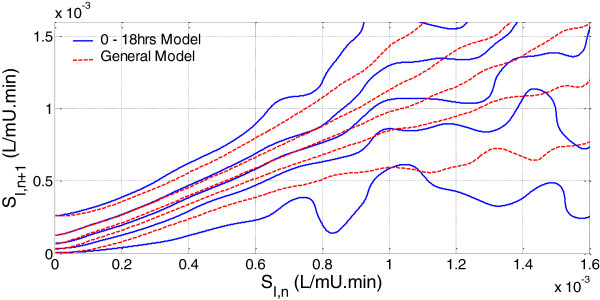
Comparison of 0–18 hrs and general stochastic models where the lines represent the 5th, 25th, 50th, 75th and 95th percentiles from the x-axis upward.

Figure 3 compares the *0*–*18 hour stochastic model* with the *general model* used in this analysis. The y-axis shows the distribution of expected SI values for hour n + 1 *given* the x-axis value of SI at hour n. The lines on this plot represent the 5^th^, 25^th^, 50^th^, 75^th^ and 95^th^ percentiles. The *0*–*18 hour model* has noticeably wider intervals between the 5^th^ – 95^th^ percentiles than the *general model*. This increased width is expected due to the increased hour-to-hour patient variability during the first 18 hours of patient stay and reflects the results presented in Figure 2A and D. Additionally, data points for the *0*–*18 hour model* are concentrated at lower SI levels, also matching the previously reported results.

**Figure 4:**
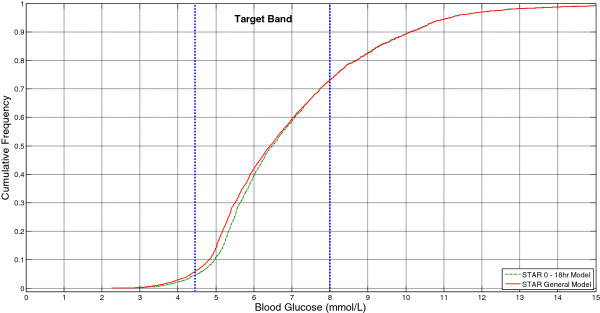
Cumulative distribution of the BG results from the virtual trial simulations performed over the first 18 hours of ICU stay using a specific 0 -18 hr stochastic model and the general model, with the STAR protocol.

Figure 4 and Table 3 summarise the results of the virtual trials comparing use of a specific *0*–*18 hour stochastic model* with the *general model*. These results show a shift in the BG distribution at low BG levels (<7 mmol/L) when compared to the *general model*. This result fits with the wider percentile bands of the *0*–*18 hour model*, and represents a small reduction in overall cohort glycaemic variability. Despite this shift, the median BG for the *0*–*18 hour model* is only 0.2 mmol/L higher than the *general model* and is still well within the target band shown in Figure 4. The interquartile range of the per-patient median glycaemia is reduced using the *0*–*18 hour model*, demonstrating the benefit of using this separate stochastic model during a known period of patient variability.

**Table 3:** Virtual trial simulation BG results comparison for data within 18 hours of ICU admission

**Whole cohort control statistics**	**STAR general model**	**STAR 2 0 -18 hrs model**
Num Patients	287	287
Total hours	5591 hours	5292 hours
Num BG measurements	4232	4096
Target BG band (mmol/L)	4.4 – 8.0	4.4 - 8.0
BG median [IQR] (mmol/L)	6.3 [5.3 - 8.1]	6.5 [5.5 - 8.1]
% BG within 4.0 - 6.1 mmol/L	45.9	42.9
% BG within 4.4 - 7.0 mmol/L	55.4	54.7
% BG within 4.4 - 8.0 mmol/L	68.7	69.0
% BG within 8.0 - 10 mmol/L	15.7	16.1
% BG > 10 mmol/L	10.2	10.5
% BG < 4.4 mmol/L	5.5	4.4
% BG < 4.0 mmol/L	3.0	2.2
% BG < 3 mmol/L	0.3	0.2
Num patients < 2.2 mmol/L	0	0

Importantly, the *0*–*18 hour stochastic model* reduces the number of hypoglycaemic measurements. For time spent below 4.4, 4.0 and 3.0 mmol/L absolute reductions of 1.1%, 0.8% and 0.1% were achieved, respectively (relative reductions of 20-33%). These values represent clinically significant changes (p = 0.008, p = 0.0098 and p = 0.35, Two-Tailed Fishers Exact Test, respectively), particularly given the association of hypoglycaemia with negative patient outcome [11]. No severe hypoglycaemic events (BG < 2.2 mmol/L) occurred for either case.

## Discussion

The use of a time-specific *0*–*18 hour stochastic model* reduces hypoglycaemia and glycaemic variability over the first 18 hours of patient admission. This reduction in hypoglycaemia is due to the wider percentile bands of the *0*–*18 hour stochastic model*, as seen in Figure 3. The STAR protocol constrains the 5^th^ percentile BG prediction to a lower limit, and thus increased width of the percentile bands causes a shift in the BG distribution to higher levels.

Approximately 300–400 patients a year receive glycaemic control from the STAR protocol in Christchurch hospital Intensive Care Unit. These patients are typically treated by the STAR protocol for approximately 5 days, resulting in 24000 BG measurements per year. Hence, a reduction of BG < 4.4 mmol/L by 1.1% could decrease the number of mild-hypoglycaemic events by up to 260 each year.

Additionally, the BG interquartile range was reduced by applying the *0*–*18 hour model*. This result indicates a small reduction on overall cohort glycaemic variability and may be due to less ‘over control’ by STAR for variable patients. Hence, these virtual trials indicate that the impact of SI variability can be reduced by using more than one stochastic model. This reduced variability is potentially beneficial as glycaemic variability has been shown to be independently associated with mortality in critically ill patients [10-13]. Therefore, not only does the additional stochastic model improve control performance by reducing the occurrence of hypoglycaemia, it also has the potential to improve patient outcomes by reducing variability.

It has been proposed that a number of factors, including cardiovascular surgery and glucocorticoid therapy, are associated with increased SI variability. Thus it would be ideal to be able to create separate stochastic models for each of these different cases, and potentially separate models for different time frames with in the first 24 hours of ICU stay. However, currently we do not have enough available data to create specific stochastic models for all proposed cases, while ensuring the data used in each is independent of the others. Nevertheless, this study has validated the concept of using additional stochastic models to achieve more accurate glycaemic control with reduced hypoglycaemia.

## Conclusions

Insulin sensitivity levels increase significantly, while variability decreases over the first few days of patient stay in the ICU. This study determined that the largest changes in SI level and variability occur during the first 18 post ICU admission, and thus, a separate stochastic model of SI behaviour for this period is warranted for use with model-based controllers to better manage this evolution.

Virtual trials using a separate stochastic model for the first 18 hours of stay demonstrated a reduction in both glycaemic variability and hypoglycaemia during this period without adversely affecting the overall level of control. Thus, use of multiple models can reduce the impact of SI variability during model-based glycaemic control. As more data becomes available this same method could be used to analyse and generate stochastic models specific to various diagnostic categories, drug therapies or other situations that are thought to increase metabolic variability.

### Key message

The impact of Insulin Sensitivity variability on glycaemic outcomes can be reduced by using separate stochastic models.

## Abbreviations

ICU: Intensive care unit; SI: Insulin sensitivity; BG: Blood glucose; ICING: Intensive control insulin-nutrition-glucose; STAR: Stochastic TARgeted; SPRINT: Specialised relative insulin nutrition tables; IQR: Interquartile range.

## Competing interests

The authors declare that they have no competing interests.

## Authors’ contributions

FT carried out the analysis of SI variability and the stochastic model generation and simulation. CP carried out an initial study in to SI variability and over saw the design and coordination of this follow up study. LF assisted with the generation of a new stochastic model and simulation of this model. GS provided clinical insight and supervised the acquisition of all clinical data used. JGC conceived of the study, and participated in its design. TD participated in the design and coordination of this study. All authors read and approved the final manuscript.
